# Diaqua­[5-(2-pyrid­yl)tetra­zolato-κ^2^ *N* ^1^,*N* ^5^]manganese(II)

**DOI:** 10.1107/S1600536808010106

**Published:** 2008-05-03

**Authors:** Xiao-Chun Wen

**Affiliations:** a Ordered Matter Science Research Centre, College of Chemistry and Chemical Engineering, Southeast University, Nanjing 210096, People’s Republic of China

## Abstract

The title compound, [Mn(C_6_H_4_N_5_)_2_(H_2_O)_2_], was synthesized by the hydro­thermal reaction of Mn(NO_3_)_2_ with picolino­nitrile in the presence of NaN_3_. The Mn atom lies on an inversion centre. The distorted octa­hedral Mn environment contains two planar *trans*-related *N*,*N*′-chelating 5-(2-pyrid­yl)­tetra­zolate ligands in the equatorial plane and two axial water mol­ecules. O—H⋯N hydrogen bonds generate an infinite three-dimensional network.

## Related literature

For the chemisty of tetra­zole, see: Arp *et al.* (2000 ▶); Dunica *et al.* (1991 ▶); Wang *et al.* (2005 ▶); Wittenberger & Donner (1993 ▶).
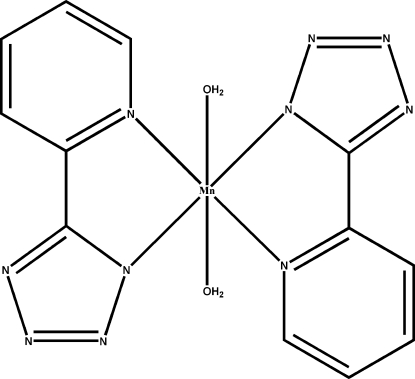


## Experimental

### 

#### Crystal data

[Mn(C_6_H_4_N_5_)_2_(H_2_O)_2_]
*M* *_r_* = 383.26
Monoclinic, 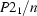

*a* = 6.185 (3) Å
*b* = 12.110 (7) Å
*c* = 10.615 (5) Å
β = 106.597 (12)°
*V* = 761.9 (7) Å^3^
*Z* = 2
Mo *K*α radiation
μ = 0.90 mm^−1^
*T* = 293 (2) K
0.5 × 0.5 × 0.4 mm

#### Data collection

Rigaku Mercury2 diffractometer
Absorption correction: multi-scan (*CrystalClear*; Rigaku, 2005 ▶) *T*_min_ = 0.638, *T*_max_ = 0.695
7656 measured reflections
1803 independent reflections
1660 reflections with *I* > 2σ(*I*)
*R*_int_ = 0.021

#### Refinement

*R*[*F* ^2^ > 2σ(*F* ^2^)] = 0.028
*wR*(*F* ^2^) = 0.071
*S* = 1.10
1803 reflections
115 parameters
H-atom parameters constrained
Δρ_max_ = 0.26 e Å^−3^
Δρ_min_ = −0.30 e Å^−3^

### 

Data collection: *CrystalClear* (Rigaku, 2005 ▶); cell refinement: *CrystalClear*; data reduction: *CrystalClear*; program(s) used to solve structure: *SHELXS97* (Sheldrick, 2008 ▶); program(s) used to refine structure: *SHELXL97* (Sheldrick, 2008 ▶); molecular graphics: *ORTEPIII* (Burnett & Johnson, 1996 ▶), *ORTEP3* (Farrugia, 1997 ▶) and *SHELXTL* (Sheldrick, 2008 ▶); software used to prepare material for publication: *SHELXL97*.

## Supplementary Material

Crystal structure: contains datablocks I, global. DOI: 10.1107/S1600536808010106/dn2338sup1.cif

Structure factors: contains datablocks I. DOI: 10.1107/S1600536808010106/dn2338Isup2.hkl

Additional supplementary materials:  crystallographic information; 3D view; checkCIF report

## Figures and Tables

**Table 1:** Hydrogen-bond geometry (Å, °)

*D*—H⋯*A*	*D*—H	H⋯*A*	*D*⋯*A*	*D*—H⋯*A*
O1*W*—H2*W*⋯N3^i^	0.85	2.03	2.864 (2)	169
O1*W*—H1*W*⋯N5^ii^	0.86	1.93	2.788 (2)	175

Symmetry codes: (i) 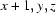; (ii) 
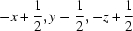
.

**Fig. 1.**
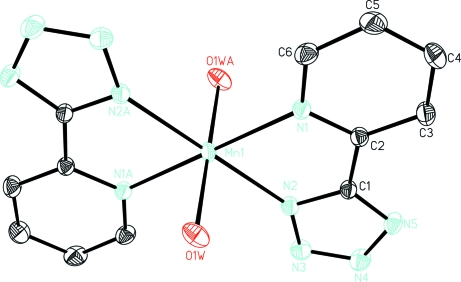
Molecular view of the title compound with the atomic numbering scheme. Displacement ellipsoids are drawn at the 50% probability level. H atoms have been omitted for clarity. [Symmetry code : (i) 1-x, 1-y, 1-z]

**Fig. 2.**
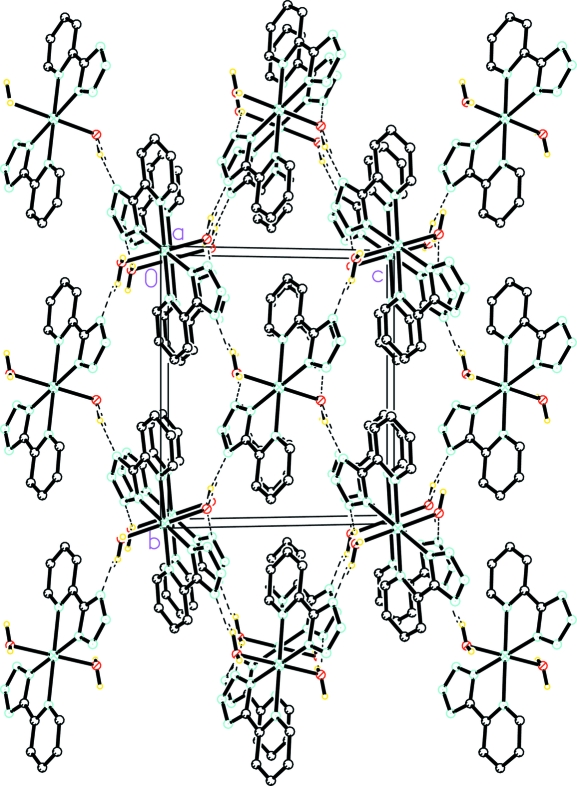
The crystal packing of the title compound viewed along the a axis showing the three dimensionnal hydrogen bondings network (dashed line). Hydrogen atoms not involved in hydrogen bonding have been omitted for clarity.

## supplementary crystallographic information

### Comment

The tetrazole functional group has found a wide range of applications in coordination chemistry as ligands, in medicinal chemistry as a metabolically stable surrogate for a carboxylic acid group, and in materials science as high density energy materials(Wang *et al.*, 2005; Dunica *et al.*, 1991; Wittenberger & Donner, 1993). We report here the crystal structure of the title compound, 5-(2-pyridyl)tetrazolate-Manganese(II) dihydrate.

The Mn atom lies on an inversion centre. The distorted octahedral Mn environment contains two planar *trans*-related *N*,*N*-chelating 5-(2-pyridyl)tetrazolate ligands in the equatorial plane and two water molecules ligands. The pyridine and tetrazole rings are nearly coplanar and are twisted from each other by a dihedral angle of only 4.02 (0.09) °(Fig.1). The bond distances and bond angles of the tetrazole rings are in the usual ranges (Wang *et al.*, 2005; *Arp *et al.**, 2000).

The O atoms from water molecules are involved in intermolecular hydrogen bonds building up an infinite three-dimensional network(Table 1, Fig. 2).

### Experimental

A mixture of picolinonitrile (0.2 mmol), NaN_3_ (0.4 mmol), Mn(NO_3_)_2_(0.15 mmol) ethanol (1 ml) and a few drops of water sealed in a glass tube was maintained at 120 °C. Yellow block crystals suitable for X-ray analysis were obtained after 3 days.

### Refinement

All H atoms attached to C atoms were fixed geometrically and treated as riding with C—H = 0.93 Å and U_iso_(H) = 1.2U_eq_(C). H atoms of water molecule were located in difference Fourier maps and included in the subsequent refinement using restraints (O-H= 0.85 (1)Å and H···H= 1.49 (2)Å) with U_iso_(H) = 1.5U_eq_(O). In the last stage of refinement they were treated as riding on the O atom.

### Crystal data

**Table:**

[Mn(C_6_H_4_N_5_)_2_(H_2_O)_2_]	*F*_000_ = 390
*M**_r_* = 383.26	*D*_x_ = 1.671 Mg m^−^^3^
Monoclinic, *P*2_1_/*n*	Mo *K*α radiation λ = 0.71073 Å
Hall symbol: -P 2yn	Cell parameters from 2090 reflections
*a* = 6.185 (3) Å	θ = 3.8–27.5º
*b* = 12.110 (7) Å	µ = 0.90 mm^−^^1^
*c* = 10.615 (5) Å	*T* = 293 (2) K
β = 106.597 (12)º	Block, yellow
*V* = 761.9 (7) Å^3^	0.5 × 0.5 × 0.4 mm
*Z* = 2	

### Data collection

**Table:**

Rigaku Mercury2 (2x2 bin mode) diffractometer	1803 independent reflections
Radiation source: fine-focus sealed tube	1660 reflections with *I* > 2σ(*I*)
Monochromator: graphite	*R*_int_ = 0.021
Detector resolution: 13.6612 pixels mm^-1^	θ_max_ = 27.9º
*T* = 293(2) K	θ_min_ = 2.6º
ω scans	*h* = −8→8
Absorption correction: multi-scan(CrystalClear; Rigaku, 2005)	*k* = −15→15
*T*_min_ = 0.638, *T*_max_ = 0.695	*l* = −13→13
7656 measured reflections	

### Refinement

**Table:**

Refinement on *F*^2^	Secondary atom site location: difference Fourier map
Least-squares matrix: full	Hydrogen site location: inferred from neighbouring sites
*R*[*F*^2^ > 2σ(*F*^2^)] = 0.028	H-atom parameters constrained
*wR*(*F*^2^) = 0.071	*w* = 1/[σ^2^(*F*_o_^2^) + (0.0346*P*)^2^ + 0.2477*P*] where *P* = (*F*_o_^2^ + 2*F*_c_^2^)/3
*S* = 1.11	(Δ/σ)_max_ = 0.001
1803 reflections	Δρ_max_ = 0.26 e Å^−^^3^
115 parameters	Δρ_min_ = −0.30 e Å^−^^3^
Primary atom site location: structure-invariant direct methods	Extinction correction: none

### Special details

**Table:**

Geometry. All esds (except the esd in the dihedral angle between two l.s. planes) are estimated using the full covariance matrix. The cell esds are taken into account individually in the estimation of esds in distances, angles and torsion angles; correlations between esds in cell parameters are only used when they are defined by crystal symmetry. An approximate (isotropic) treatment of cell esds is used for estimating esds involving l.s. planes.
Refinement. Refinement of *F*^2^ against ALL reflections. The weighted *R*-factor *wR* and goodness of fit *S* are based on *F*^2^, conventional *R*-factors *R* are based on *F*, with *F* set to zero for negative *F*^2^. The threshold expression of *F*^2^ > σ(*F*^2^) is used only for calculating *R*-factors(gt) *etc*. and is not relevant to the choice of reflections for refinement. *R*-factors based on *F*^2^ are statistically about twice as large as those based on *F*, and *R*- factors based on ALL data will be even larger.

### Fractional atomic coordinates and isotropic or equivalent isotropic displacement parameters (Å^2^)

**Table:**

	*x*	*y*	*z*	*U*_iso_*/*U*_eq_	
C1	0.2334 (2)	0.69220 (11)	0.35475 (13)	0.0241 (3)	
C2	0.4533 (2)	0.74376 (11)	0.41494 (13)	0.0244 (3)	
C3	0.4930 (3)	0.85522 (12)	0.40133 (15)	0.0344 (3)	
H3	0.3799	0.9008	0.3511	0.041*	
C4	0.7043 (3)	0.89713 (13)	0.46408 (17)	0.0412 (4)	
H4	0.7349	0.9717	0.4569	0.049*	
C5	0.8696 (3)	0.82755 (14)	0.53750 (16)	0.0393 (4)	
H5	1.0121	0.8544	0.5814	0.047*	
C6	0.8179 (2)	0.71703 (13)	0.54410 (15)	0.0325 (3)	
H6	0.9299	0.6698	0.5921	0.039*	
Mn1	0.5000	0.5000	0.5000	0.02576 (11)	
N1	0.61452 (19)	0.67490 (9)	0.48475 (11)	0.0255 (2)	
N2	0.19348 (18)	0.58625 (9)	0.37478 (12)	0.0275 (2)	
N3	−0.0219 (2)	0.56898 (11)	0.30475 (13)	0.0338 (3)	
N4	−0.1061 (2)	0.66105 (11)	0.24559 (13)	0.0368 (3)	
N5	0.0515 (2)	0.74084 (10)	0.27584 (12)	0.0318 (3)	
O1W	0.58488 (19)	0.45145 (9)	0.32058 (11)	0.0393 (3)	
H1W	0.5510	0.3858	0.2905	0.059*	
H2W	0.7096	0.4775	0.3151	0.059*	

### Atomic displacement parameters (Å^2^)

**Table:**

	*U*^11^	*U*^22^	*U*^33^	*U*^12^	*U*^13^	*U*^23^
C1	0.0229 (6)	0.0215 (6)	0.0268 (6)	0.0016 (5)	0.0055 (5)	0.0021 (5)
C2	0.0237 (6)	0.0221 (6)	0.0267 (6)	−0.0007 (5)	0.0059 (5)	0.0014 (5)
C3	0.0350 (7)	0.0238 (7)	0.0409 (8)	−0.0016 (6)	0.0050 (6)	0.0051 (6)
C4	0.0445 (9)	0.0267 (7)	0.0491 (9)	−0.0124 (6)	0.0082 (7)	0.0013 (6)
C5	0.0299 (7)	0.0422 (9)	0.0407 (8)	−0.0135 (6)	0.0019 (6)	−0.0014 (7)
C6	0.0243 (6)	0.0366 (8)	0.0327 (7)	−0.0011 (6)	0.0020 (5)	0.0024 (6)
Mn1	0.02613 (16)	0.01760 (16)	0.03133 (17)	0.00182 (10)	0.00463 (12)	0.00246 (10)
N1	0.0235 (5)	0.0237 (5)	0.0277 (5)	0.0000 (4)	0.0047 (4)	0.0021 (4)
N2	0.0222 (5)	0.0227 (6)	0.0342 (6)	−0.0018 (4)	0.0026 (5)	0.0001 (4)
N3	0.0239 (6)	0.0332 (7)	0.0400 (7)	−0.0042 (5)	0.0023 (5)	−0.0009 (5)
N4	0.0246 (6)	0.0402 (7)	0.0407 (7)	−0.0016 (5)	0.0012 (5)	0.0044 (6)
N5	0.0240 (6)	0.0316 (6)	0.0363 (6)	0.0023 (5)	0.0029 (5)	0.0075 (5)
O1W	0.0422 (6)	0.0340 (6)	0.0472 (6)	−0.0123 (5)	0.0213 (5)	−0.0121 (5)

### Geometric parameters (Å, °)

**Table:**

C1—N5	1.3325 (17)	C6—H6	0.9300
C1—N2	1.3350 (18)	Mn1—O1W^i^	2.1954 (14)
C1—C2	1.4670 (19)	Mn1—O1W	2.1954 (14)
C2—N1	1.3478 (17)	Mn1—N2^i^	2.2388 (14)
C2—C3	1.387 (2)	Mn1—N2	2.2388 (14)
C3—C4	1.383 (2)	Mn1—N1^i^	2.2538 (16)
C3—H3	0.9300	Mn1—N1	2.2538 (16)
C4—C5	1.381 (2)	N2—N3	1.3438 (17)
C4—H4	0.9300	N3—N4	1.3122 (19)
C5—C6	1.382 (2)	N4—N5	1.3449 (18)
C5—H5	0.9300	O1W—H1W	0.8597
C6—N1	1.3367 (18)	O1W—H2W	0.8507
			
N5—C1—N2	111.33 (12)	N2^i^—Mn1—N2	180.0
N5—C1—C2	126.65 (12)	O1W^i^—Mn1—N1^i^	91.80 (5)
N2—C1—C2	122.03 (11)	O1W—Mn1—N1^i^	88.20 (5)
N1—C2—C3	122.30 (13)	N2^i^—Mn1—N1^i^	75.47 (5)
N1—C2—C1	115.11 (12)	N2—Mn1—N1^i^	104.53 (5)
C3—C2—C1	122.59 (12)	O1W^i^—Mn1—N1	88.20 (5)
C4—C3—C2	118.56 (14)	O1W—Mn1—N1	91.80 (5)
C4—C3—H3	120.7	N2^i^—Mn1—N1	104.53 (5)
C2—C3—H3	120.7	N2—Mn1—N1	75.47 (5)
C5—C4—C3	119.57 (14)	N1^i^—Mn1—N1	180.0
C5—C4—H4	120.2	C6—N1—C2	118.14 (12)
C3—C4—H4	120.2	C6—N1—Mn1	126.70 (10)
C4—C5—C6	118.32 (14)	C2—N1—Mn1	115.00 (9)
C4—C5—H5	120.8	C1—N2—N3	105.11 (11)
C6—C5—H5	120.8	C1—N2—Mn1	112.29 (9)
N1—C6—C5	123.09 (14)	N3—N2—Mn1	142.51 (9)
N1—C6—H6	118.5	N4—N3—N2	109.13 (12)
C5—C6—H6	118.5	N3—N4—N5	109.55 (12)
O1W^i^—Mn1—O1W	180.0	C1—N5—N4	104.88 (12)
O1W^i^—Mn1—N2^i^	88.95 (5)	Mn1—O1W—H1W	118.4
O1W—Mn1—N2^i^	91.05 (5)	Mn1—O1W—H2W	114.0
O1W^i^—Mn1—N2	91.05 (5)	H1W—O1W—H2W	116.5
O1W—Mn1—N2	88.95 (5)		

Symmetry codes: (i) −*x*+1, −*y*+1, −*z*+1.

### Hydrogen-bond geometry (Å, °)

**Table:**

*D*—H···*A*	*D*—H	H···*A*	*D*···*A*	*D*—H···*A*
O1W—H2W···N3^ii^	0.85	2.03	2.864 (2)	169
O1W—H1W···N5^iii^	0.86	1.93	2.788 (2)	175

Symmetry codes: (ii) *x*+1, *y*, *z*; (iii) −*x*+1/2, *y*−1/2, −*z*+1/2.

## References

[bb1] Arp, H. P. H., Decken, A., Passmore, J. & Wood, D. J. (2000). *Inorg. Chem.* **39**, 1840–1848. 10.1021/ic990828q 11428102

[bb2] Burnett, M. N. & Johnson, C. K. (1996). *ORTEPIII* Report ORNL-6895. Oak Ridge National Laboratory, Tennessee, USA.

[bb3] Dunica, J. V., Pierce, M. E. & Santella, J. B. III (1991). *J. Org. Chem.* **56**, 2395–2400.

[bb4] Farrugia, L. J. (1997). *J. Appl. Cryst.* **30**, 565.

[bb5] Rigaku (2005). *CrystalClear* Rigaku Corporation, Tokyo, Japan.

[bb6] Sheldrick, G. M. (2008). *Acta Cryst.* A**64**, 112–122. 10.1107/S0108767307043930 18156677

[bb7] Wang, X.-S., Tang, Y.-Z., Huang, X.-F., Qu, Z.-R., Che, C.-M., Chan, C. W. H. & Xiong, R.-G. (2005). *Inorg. Chem.* **44**, 5278–5285. 10.1021/ic050354x 16022526

[bb8] Wittenberger, S. J. & Donner, B. G. (1993). *J. Org. Chem.* **58**, 4139–4141.

